# The Vasoreparative Potential of Endothelial Colony Forming Cells: A Journey Through Pre-clinical Studies

**DOI:** 10.3389/fmed.2018.00273

**Published:** 2018-10-16

**Authors:** Christina L. O'Neill, Kiran J. McLoughlin, Sarah E. J. Chambers, Jasenka Guduric-Fuchs, Alan W. Stitt, Reinhold J. Medina

**Affiliations:** Centre for Experimental Medicine, Queen's University Belfast, Belfast, United Kingdom

**Keywords:** endothelial progenitor cells (EPC), angiogenesis, cell therapy, endothelial colony forming cells (ECFCs), ischemia, vascular repair

## Abstract

For over a decade various cell populations have been investigated for their vasoreparative potential. Cells with the capacity to promote blood vessel regeneration are commonly known as endothelial progenitor cells (EPCs); although such a definition is currently considered too simple for the complexity of cell populations involved in the reparative angiogenic process. A subset of EPCs called endothelial colony forming cells (ECFCs) have emerged as a suitable candidate for cytotherapy, primarily due to their clonogenic progenitor characteristics, unequivocal endothelial phenotype, and inherent ability to promote vasculogenesis. ECFCs can be readily isolated from human peripheral and cord blood, expanded *ex vivo* and used to revascularize ischemic tissues. These cells have demonstrated efficacy in several *in vivo* preclinical models such as the ischemic heart, retina, brain, limb, lung and kidney. This review will summarize the current pre-clinical evidence for ECFC cytotherapy and discuss their potential for clinical application.

## The potential of vascular regeneration as a therapeutic goal

Vasodegeneration and the ensuing tissue ischemia remains a significant challenge for healthcare systems worldwide. Diseases such as ischemic heart disease, stroke, peripheral artery disease, and ischemic retinopathies are complex to treat because there are diverse underpinning causes of non-perfusion and each tissue exhibits a distinct response to hypoxia. Current available treatments aim to remove damaged tissue, widen obstructed blood vessels or replace blocked vasculature with bypass surgery. A potential new approach would be to regenerate the compromised vasculature using so-called “therapeutic angiogenesis”. While delivery of pro-angiogenic peptides have often provided scope for achieving re-perfusion, the use of cell therapy products have gained favor since they can offer sustained delivery of a multitude of angiogenic factors and/or provide direct replacement of damaged vascular cells (1). Although several cell-types such as mesenchymal stem cells (MSCs) (2), embryonic stem cells (ESCs) (3), and induced pluripotent stem cells (iPSCs) (4) have been tested pre-clinically for their therapeutic potential, endothelial progenitor cells (EPCs) have emerged as a population of cells with promising tissue regenerative properties.

EPCs have been the subject of considerable controversy due to their ambiguous definition (5) and this term actually encompasses a range of very different cell types (6). Cells named as myeloid angiogenic cells (MACs), circulating angiogenic cells (CACs), and early EPCs, all of which are hematopoietic cells, have been shown to promote vascular repair through paracrine mechanisms. Recently, leading experts in the field published a consensus statement on EPC nomenclature to standardize terminology (7). Endothelial progenitors are thus defined as cells with an unequivocal endothelial phenotype, self-renewal potential and a capacity for *de novo, in vivo* blood vessel formation. It is now widely accepted that endothelial colony forming cells (ECFCs) comply with this definition and are considered the “*bona fide* endothelial progenitor” with emerging therapeutic potential (7).

## What are ECFCs?

**Figure 1:**
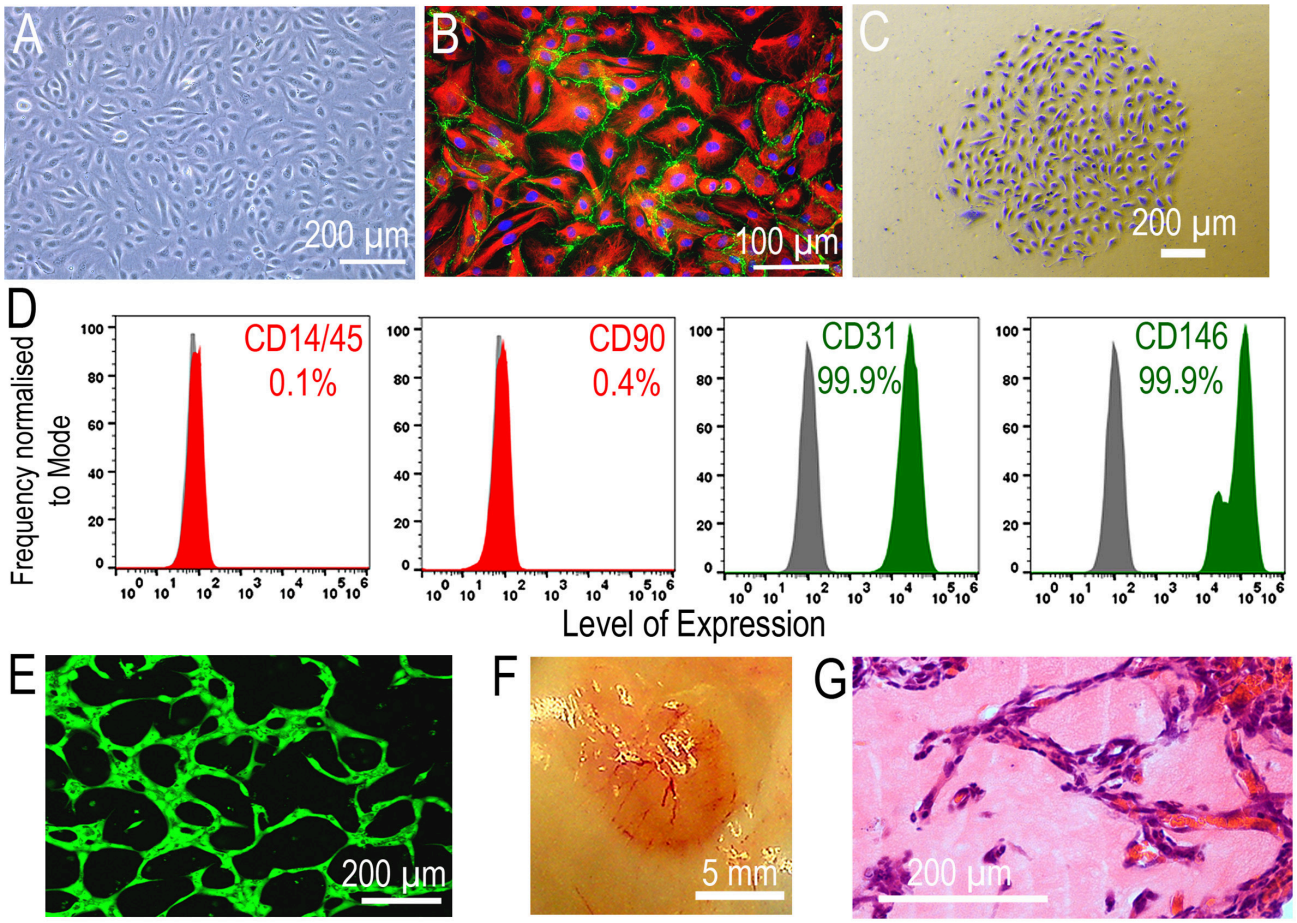
Properties of ECFCs. **(A)** ECFC cobblestone monolayer morphology (Scale bar 200 μm). **(B)** A tight monolayer of ECFCs with adherens junctions (β catenin = green, Vimentin = red, scale bar 100 μm). **(C)** A colony derived from a single cell demonstrating clonogenic potential of ECFCs (crystal violet stained, scale bar 200 μm). **(D)** ECFCs express endothelial markers CD31 & CD146 and are negative for hematopoietic markers CD14/45 and stromal marker CD90. **(E)** ECFCs have tubulogenic capacity *in vitro* (scale bar 200 μm) **(F,G)**. ECFCs form perfused vessels *in vivo* in the Matrigel plug assay (Scale bar, 5 mm and 200 μm respectively).

Endothelial colony forming cells (ECFCs) are sometimes referred to in the literature as late Endothelial Progenitor cells (due to their later appearance in culture), blood outgrowth endothelial cells, or outgrowth endothelial cells. ECFCs are isolated *in vitro* from the cultured mononuclear fraction of peripheral blood or umbilical cord blood, grown under endothelial conditions. They appear in culture as cobblestone shaped colonies within weeks of mononuclear cell plating and have significant proliferative potential (Figure 1) (8). It has been demonstrated that ECFCs can also be derived from human induced pluripotent stem cells by sorting for markers Neuropilin-1 and CD31 (9). In addition to cord blood, ECFCs have also been successfully isolated from fat tissue (10), placenta (11), and lungs (12); these findings suggest that ECFCs originate from tissue resident vascular progenitors. Recent reports pinpoint specific endothelial subsets within the vasculature and these may constitute “vascular stem cells” with homeostatic reparative roles. These vascular stem cells are identified by the expression of CD201, the protein C receptor (PROCR) EPCR, a type 1 transmembrane receptor which is known to be highly expressed on vascular endothelial stem cells (VESCs). PROCR+ selection facilitates their isolation and enriches for highly clonogenic cells with bipotent differentiation capacity into endothelial cells and pericytes (13). CD157, also known as bone marrow stromal antigen 1 has also been identified as a marker of tissue resident VESCs, it is expressed in endothelial cells of large vessels and CD157+ cells possess significant vascular regenerative potential (14).

## The hallmarks of ECFCs

ECFCs typically exhibit high clonogenic capacity. Indeed, these cells can yield a hierarchy of different sized colonies with umbilical cord blood giving rise to the highest frequency of largest colonies that have high proliferative potential (HPP) when compared to adult peripheral blood (15). ECFCs are characterized by the expression of endothelial markers CD31, CD146, VEGFR2, vWF, and VE-cadherin. ECFCs also express CD34, although the frequency of this marker is variable and can diminish as the cells are expanded *in vitro*. Importantly, ECFCs are negative for hematopoietic markers CD14 and CD45. An essential property of ECFCs is their ability to form either a *de novo* vascular network *in vitro* while *in vivo* these cells integrate seamlessly with the host vasculature (Figure 1).

## Pre-clinical application of ECFCs

### The ischemic retina

Ischemic retinopathies such as retinal vein occlusion, diabetic retinopathy, and retinopathy of prematurity are common causes of visual impairment and are characterized by vasodegeneration (16). Pre-clinical evidence shows that ECFC cell therapy may be a potential treatment strategy for such ischemic retinopathies (17). The retina differs from other organs, as it has a certain degree of immune privilege and so provides a unique environment to examine the effects of human ECFCs. When these cells were injected into murine models of retinal ischemia, they promoted vascular repair, decreased the avascular area, enhanced the normovascular area, and importantly, decreased pathological neovascular tuft formation. Furthermore, human ECFCs could be found directly integrating and forming new vessels within the host murine vasculature (8). The same effects were seen when ECFCs derived from induced pluripotent stem cells were used (9). In addition, beneficial effects of ECFCs may be enhanced using agents that alter growth factor signaling pathways. For example, AAV2.COMP-Ang1 was shown to enhance the therapeutic benefit of intravitreally delivered ECFCs by promoting their integration into the murine vasculature (18). Although there is a lot more work needed before translation of ECFCs into therapy for the ischemic retina, we have recently evaluated the effect of ECFCs in the mouse oxygen-induced retinopathy model, by examining dose, delivery route, and immunogenicity. Human ECFCs delivered to the murine ischemic retina demonstrated a vasoreparative effect both by intravitreal and intracarotid delivery. Importantly, cells conferred therapeutic benefit by promoting vascular repair. In addition, if ECFCs were delivered into healthy adult mice, they were completely cleared from the retina within 3 days (17). It has also been reported that ECFCs promote vascular repair in the ischemic retina through release of paracrine factors. A subset of ECFCs was found to be effective in rescuing retinal degeneration, this subset was found to express high levels of CD44, the hyaluronic acid receptor (19). Taken together, these data provide convincing scientific evidence to support ECFCs as a potential cell therapy for ischemic retinopathies.

### The ischemic brain

Ischemic stroke is a common worldwide cause of mortality. Fewer than 10% of patients experiencing ischemic stroke are suitable for thrombolysis treatment which can effectively restore some cerebral blood flow (20), therefore there is a need for therapies that induce vascular repair and more effectively restore blood flow for greater number of patients. ECFCs are emerging as a promising new treatment option for ischemic stroke and their therapeutic potential in the rodent brain has been demonstrated. For example, GFP-labeled ECFCs were tracked for cell engraftment in a photothrombotic ischemic stroke mouse model. Cells were delivered via the left cardiac ventricle 24 h after stroke. Bioluminescence signals were highest in the brain on day 1 and decreased steadily until day 14. GFP-positive ECFCs were found at the infarct border demonstrating a successful homing response to regions of tissue hypoxia. Importantly, ECFC therapy led to improved angiogenesis, neurogenesis, decreased neuronal apoptosis, and ultimately led to restoration of brain function (21). In addition, in a rat model of transient focal cerebral ischemia and middle cerebral artery occlusion (MCAO), ECFCs primed with erythropoietin (EPO) were shown to have enhanced efficacy for reversing stroke injury (22, 23). In another study using the same model, labeled ECFCs administered intravenously 24 h after MCAO were seen to specifically home to the ischemic hemisphere and settled in the injured area. ECFCs transplantation stimulated an increase in capillary density at the site of injury. Although in this study, ECFCs were not detected integrating within the vasculature, they stimulated an increase in proangiogenic growth factor expression at the ischemic site, which was also associated with a reduction in the number of apoptotic cells (24).

ECFCs have also been examined for their potential to repair vascular damage in pre-clinical models of traumatic brain injury (TBI) in rodents. TBI, created by lateral fluid percussion injury was used to assess the effects of cord blood-derived ECFCs. Cells were intravenously infused 1 h after injury. ECFCs successfully homed to the injured site, were detected in the injured brain after 24 h and were effective in promoting neovascularization and improving neurological function (25). A further follow-up study demonstrated that infusion of ECFCs can repair blood brain barrier tight junction functionality (26). In addition, in a rat model of cerebral aneurysm, ECFC transfusion was shown to inhibit inflammatory signaling, protect smooth muscle cells from apoptosis and promote vascular regeneration (27). Taken together, these studies highlight the potential therapeutic effects of ECFCs for vascular repair in the brain.

### Peripheral artery disease

Peripheral artery disease (PAD) can lead to ischemic injury and amputation. Prognosis is poor and current treatments for PAD patients are limited (50); therefore there is a pressing need for new strategies to enhance angiogenesis and collateral arterial growth. The potential for ECFC treatment in PAD has been demonstrated in several preclinical studies using the murine hindlimb ischemia model. Firstly, ECFCs were shown to increase perfusion by rapidly relocating to the ischemic hind limb within 6 h after injection. In addition, there was enhanced benefit when a combination of ECFCs and mesenchymal stem cells (MSCs) were used. Further analysis showed that the reparative effects of ECFC therapy were due to direct vascular incorporation (28). Although it has also been suggested that ECFCs can function as paracrine mediators, modulating MSC engraftment via PDGF-BB/PDGFRb signaling (29). Similar beneficial effects were reported in a later study in which ECFCs combined with mesenchymal progenitor cells enhanced blood flow recovery of the ischemic limb. Interestingly, analysis showed that improved blood flow was in part due to the recruitment of host myeloid cells with, presumably, concomitant release of pro-angiogenic growth factors (30). Another study combining ECFCs and MSCs, via retro-orbital delivery, demonstrated the homing ability of the cells and increased vessel density via an endoglin-dependent mechanism (31). Modification or pre-treatment of ECFCs has been shown to enhance their effects. For example, ECFC-induced functional recovery and limb salvage were markedly improved by Fucoidan pre-treatment, which protected the cells from replicative cellular senescence (32). Overexpression of integrin B1 was also reported to augment ECFC vasoreparative potential as it improved homing of the cells to the ischemic tissue, leading to improved perfusion in the ischemic limbs (33). Similarly, adiponectin pre-treatment has been demonstrated to increase ECFCs neovascularization capacity in a hind limb model in streptozotocin-induced hyperglycemic nu/nu mice (34). ARA290, an agonist of Erythropoietin has also been shown to enhance ECFC function by increasing the homing of these cells to the ischemic limb (35). In addition, ECFCs derived from human induced pluripotent stem cells (iPSCs) have also been shown to contribute to hind limb vascular repair. In a model of hindlimb femoral vessel removal in nude mice, hiPSC ECFCs were shown to improve blood perfusion and limb salvage as well as cord blood-derived ECFCs (9). This result is important as it means that it may be possible to generate patient specific hiPSC-derived ECFCs for autologous treatment of vascular disease.

### The ischemic myocardium

Ischemic heart disease is a common cause of mortality worldwide. Cell therapy to regenerate the ischemic heart is a rapidly emerging concept. Most clinical trials have used bone marrow mononucleated cells (MNCs). Although bone marrow MNCs have demonstrated some therapeutic efficacy for the ischemic myocardium (51), they remain a very heterogeneous population of cells and therefore it is difficult to decipher the individual contributions of cell populations responsible for the repair. In addition, bone marrow MNCs from patients with ischemic heart disease have been shown to have a reduced neovascularization capacity (52). ECFCs remain to be tested clinically in the ischemic myocardium. The first pre-clinical studies were performed using CD34+ cells with presumed endothelial precursor capacity. These cells increased vessel density within the infarct area and improved left ventricular function although whether these heterogeneous cells were acting as paracrine reservoirs or endothelial “building blocks” remains unknown (36). Further pre-clinical studies have shown that ECFC therapy is beneficial; cells participate in the vascular repair process and promote vascular recovery of the ischemic heart. In an acute myocardial infarction model in pigs, infusion of autologous ECFC-like cells improved myocardial remodeling; decreased infarct size, increased vessel density and ECFCs were seen to incorporate within vessels at the border zone (37). Myocardial injection of ECFCs expressing AKT/HO-1 into a murine myocardial infarction model demonstrated an increase in blood vessel density, a decrease in apoptotic cells at the infarct site, reduction in oxidative stress and pro inflammatory molecule TNFα as well as an improvement in ejection fraction (38). More recently, the transplantation of ECFCs pre-treated with Genistein was shown to increase cellular proliferation at the ischemic sites, enhance neovascularization, decrease myocardial fibrosis, and improve cardiac function (39).

### Wound healing

The inability to efficiently repair wounds is a common feature of patients suffering with vascular disease. Due to their angiogenic potential, ECFCs have been examined for their capacity to aid vascular repair in several wound injury models. In a murine model where tissue injury was induced by dye laser, digital intravital microscopy revealed that human umbilical cord blood-derived ECFCs delivered via infusion through the jugular vein migrate to sites of injury and promote endothelial regeneration (40). Interestingly, this study found that recruitment of ECFCs was dependent on the presence of neutrophils at the site of injury via the binding of P-selectin glycoprotein ligand-1 (PSGL-1) (40). In a murine model of full thickness dermal wound in athymic nude mice, unlike HUVECs, treatment with ECFCs was shown to induce wound vascularization by direct integration with host vasculature, demonstrated by blood filled vasculature. Furthermore, cells were detectable up until day 10, wounds that received ECFCs showed reduced levels of hypoxia, enhanced matrix organization and accelerated epithelial coverage. Interestingly, the border of the ECFC treated wounds contained smooth muscle cells likely mobilized by the secretion of PIGF and PDGF-BB by the ECFCs. This study also demonstrated ECFCs pro-angiogenic potential by paracrine factors: ECFCs expressed higher levels of pro-angiogenic growth factors such as VEGF, PIGF, and Ang-1 compared to HUVECs; ECFC-conditioned medium (CM) significantly improved collagen matrix organization from human dermal fibroblast sheets; and ECFC-CM boosted keratinocyte migration and proliferation across the wound via secretion of KGF and HGF (41). ECFCs also have the potential to be embedded within scaffolds or skin substitutes (42) where they can vascularize the scaffolds alone or in combination with accessory cells, and following subcutaneous transplantation, can anastomose within the host vasculature enabling perfusion (43). Cord blood-derived ECFCs seeded in a RGD-g-PLLA biosynthetic scaffold (designed to promote survival and retention of the cells at the wound site) enhanced dermal wound neovascularization and labeled ECFCs were seen to be retained in the wound up to a week after transplantation (44).

### The lungs

The lungs are highly vascularized organs. Recent research indicates that lung microvascular endothelium is a rich source of resident ECFCs, which contribute to normal vascular homeostasis (12, 14). Studies investigating the effects of ECFCs in the lung have shown beneficial effects. For example, in a rat injury model of oxygen-induced bronchopulmonary dysplasia, a lung disease of prematurity, human cord blood-derived ECFCs were administered through the jugular vein. ECFCs significantly improved lung compliance and the architecture of the alveoli. It also showed that ECFC therapy improved lung angiogenesis and prevented pulmonary hypertension in hyperoxia-exposed newborn mice. Treatment with ECFC-CM showed similar therapeutic effects in such models of bronchopulmonary dysplasia (45, 46). Such studies highlight that ECFCs may provide vasoreparative effects through paracrine mechanisms, in addition to direct vascular engraftment.

### The kidney

Ischemia reperfusion injury is the main cause of acute kidney injury (AKI) which results in endothelial cell loss and apoptosis, leading to reduction in peritubular capillary blood flow (53). ECFCs may facilitate the development of new treatments for AKI. The administration of ECFCs in a mouse model of ischemic AKI was shown to reduce tubular injury, renal apoptosis, and infiltration of macrophages and attenuate increases in plasma creatinine levels. Because there was little evidence of ECFCs remaining within the murine kidneys, their protective effects were primarily attributed to the release of exosomes, as injecting ECFC-derived exosomes alone protected against the multiple parameters of kidney injury (47). A follow-up study showed that specifically the microRNA miR-486-5p, present at high amounts within the ECFC exosomes, accounted for the therapeutic effects of ECFCs (48). In agreement with this, other studies have also shown that ECFCs secrete soluble factors to preserve microvascular function. Conditioned media from human cord blood-derived ECFCs offered protection in a rat model of ischemia reperfusion injury. Interestingly, ECFC-CM significantly reduced ICAM-1 expression and decreased the number of differentiated lymphocytes recruited to the kidney after renal ischemia (49). These studies suggest that ECFCs and their CM may provide a therapeutic option for the treatment of AKI.

## Future perspectives

The studies discussed in this review (Table 1), support the case for ECFCs as a potential novel endothelial cell therapy that promotes vascular repair in many different vascular beds albeit tissue specific endothelial heterogeneity. It is possible that ECFCs, as progenitors, have required plasticity to adopt organ specific endothelial characteristics. A sole mechanism of action (MOA) for ECFC beneficial effects remains unclear. In some of these studies, ECFCs facilitate vascular repair by directly integrating within the host vasculature, while in others vascular engraftment of these cells is low or completely absent and their therapeutic benefit can be explained by the paracrine release of proangiogenic growth factors. More research is needed to fully elucidate mechanisms of action, but these are likely to depend on the experimental model used. Demonstration of long-term engraftment in pre-clinical models is challenging considering ECFCs are human cells delivered into immunocompetent mice. Other factors that need to be considered for each target disease are cell dosage, delivery route, and timing of ECFC administration (1).

**Table 1:** Summary of Pre-clinical studies using ECFCs in various disease models.

	**Retina**	**Brain**	**Limb**	**Heart**	**Skin**	**Lung**	**Kidney**
Pre-clinical model	Mouse oxygen induced retinopathy	Mouse ischemic stroke Rat transient focal cerebral ischemia Rat middle cerebral artery occlusion	Mouse hindlimb ischemia	Pig myocardial infarction Mouse myocardial infarction	Mouse tissue injury Mouse full thickness dermal wound	Rat oxygen induced bronchopulmonary dysplasia	Mouse bilateral kidney I/R injury rat I/R injury
ECFC source	Human cord blood iPS-derived	Human cord blood	Human cord blood iPS-derived Human white adipose tissue	Pig peripheral blood Mouse bone marrow Human cord blood	Human cord blood	Human cord blood	Human cord blood
Delivery	intra-vitreal Intra-carotid	Intra-arterial Intra-venous	Intra-muscular Retro-orbital	Intra-arterial Intra-myocardial	Intra-jugular Embedded in scaffold & grafted onto wound site	Intra-jugular	Intra-jugular
Results	Promote vasoreperfusion Decrease avascular area & pathological neovascular tufts	Improve neurological function & blood brain barrier integrity Improve angiogenesis & neurogenesis Decrease apoptosis & inhibit inflammatory signaling	Increase perfusion & vessel density Improve limb salvage Recruit myeloid cells	Increase blood vessel density Improve left ventricular function & myocardial remodeling Decrease myocardial fibrosis, infarct size, apoptosis & oxidative stress Anti-inflammatory	Induce wound vascularization Contribute to functional vessels Reduced levels of hypoxia Enhanced matrix organization Accelerate re-epithelialization	Improve lung compliance & architecture of alveoli Preserve lung vascularity Prevents pulmonary hypertension	Attenuate I/R-induced renal injury & renal superoxide formation Prevent tubule necrosis Reduce macrophage infiltration Decrease apoptosis
Integration into host vasculature	Direct integration and formation of new blood vessels detected 72 h post-delivery	Not detected to integrate within the vasculature	Direct incorporation into vasculature detected 20 days post delivery	Engraft into host myocardium Incorporate into vessels at border zone detected 7 weeks post delivery	Integrate into host vasculature & form functional hybrid vessels detected 10 days post delivery Scaffolds embedded with ECFCs	Low engraftment in recipient lung vasculature 14 days post delivery	Barely detectable in any tissue 24 h post delivery
Paracrine Function	Promote vascular repair through release of paracrine factors	Increased expression of pro-angiogenic growth factors	Function as paracrine mediators by modulating msc engraftment	Induce secretion of angiogenic cytokines at ischemic site	Secreted factors enhance collagen matrix organization, promote migration & proliferation of keratinocytes	ECFC-CM showed similar therapeutic effects to ECFCs	Secrete exosomes as primary mediators of anti-apoptotic effect
References	(8, 9), (17–19)	(21–27)	(9), (28–35)	(36–39)	(40–44)	(45, 46)	(47–49)

To induce maximal therapeutic efficacy, it may be worthwhile considering co-transplantation of ECFCs with accessory cells such as MSCs (28, 30), smooth muscle cells (SMCs) (54), adipose tissue-derived stem cells (43) or myeloid angiogenic cells (55). Several studies have shown this to be an effective approach as it may promote long-term neovascularization by directly supporting and stabilizing ECFC-derived neovessels and also by providing proangiogenic factors to aid the regenerative process.

The majority of studies described in this review have been performed using a xeno-allogenic approach, testing human cells in mouse and rat models. However, in order to translate this work toward the clinical setting; it might be necessary to collect data from larger animal models, which may also allow an autologous approach. ECFCs have successfully been isolated from rabbits (56), dogs (57), pigs (58), sheep (59), horses (60), and monkeys (61) and there is some evidence for autologous ECFC therapy to be efficacious in pigs (37). Despite these results, when considering the use of ECFCs as a potential cytotherapy for human disease, it is likely that initial ECFC therapy may be allogeneic due to the fact that ECFCs cannot always be obtained efficiently from adult peripheral blood and those isolated from patients may be dysfunctional and therefore not optimal for cell transplantation. Another consideration is that ECFCs in human blood are very rare, and numbers are reduced with disease (62), thus it may not be possible to isolate them from every patient. Furthermore, isolation, characterization and *ex-vivo* expansion of autologous ECFCs requires 4–6 weeks which is a limiting factor for diseases with a narrow therapeutic window. Therefore, we consider ECFC allogeneic therapy as the most feasible and practical strategy in which ECFCs will be HLA typed to match patients and will be stored in cell banks as an “off-the-shelf” cell therapy product.

There is quite a high failure rate in successful translation of regenerative medicine therapies to clinical use. This is mainly due to potential cell products not passing the regulatory requirements, clinical efficacy standards, and the high manufacturing cost (63). Successful translation in the EPC field has been mainly impaired due to the use of heterogeneous populations of non-endothelial cells such as BM-MNCs, which have led to conflicting results and discourage pursue of further clinical trials (64). High cell population heterogeneity and low purity characterized the first generation of cell therapies, but next generation cell therapies require a highly pure and well-defined cell therapy product for consistency. In the EPC field, a recently published consensus on EPC nomenclature recommended accurate cell definitions and meaningful nomenclature (7).

This review shows pre-clinical evidence suggesting that ECFCs have therapeutic value for ischemic diseases. Advances in cell therapy manufacture technologies in combination with a first-in-man clinical trial are needed to facilitate the translational pathway for ECFCs into patients. Importantly, both cells GMP manufacture and clinical trial design have to align with regulatory frameworks for advanced therapy medicinal products.

Finally, before ECFCs are used in the clinical setting, protocols for their isolation, culture, and expansion must strictly adhere to GMP standard operating procedures. GMP guidelines may vary from country to country (65). In addition, xeno-free culture conditions are preferable. Replacing the use of animal products required for ECFC culture, such as fetal bovine serum and rat Collagen I are being investigated by many groups. Strategies to overcome these issues include the use of human platelet lysate (66, 67) and the manufacture of GMP synthetic basement membrane substrates (68). In addition, the use of automated cell culture facilities will enable scalable and standardized methods to create a reliable and reproducible cell therapy product.

In conclusion, harnessing the reparative angiogenic capacity of ECFCs may provide an exciting new regenerative therapy for vascular disease, however there are still challenges to overcome, and more research is warranted before these can be used in the clinic.

## Author contributions

CO and RM: conception and design; CO, RM, and AS: manuscript writing; KM, SC, and JG-F: final approval of manuscript; CO, RM, KM, and SC: figure and table design.

### Conflict of interest statement

The authors declare that the research was conducted in the absence of any commercial or financial relationships that could be construed as a potential conflict of interest.
